# Zein nanofibers via deep eutectic solvent electrospinning: tunable morphology with super hydrophilic properties

**DOI:** 10.1038/s41598-020-72337-4

**Published:** 2020-09-17

**Authors:** Muzamil Khatri, Zeeshan Khatri, Sofia El-Ghazali, Nadir Hussain, Umair Ahmed Qureshi, Shunichi Kobayashi, Farooq Ahmed, Ick Soo Kim

**Affiliations:** 1 Nano Fusion Technology Research Group, Division of Frontier Fibers, Institute for Fiber Engineering (IFES), Interdisciplinary Cluster for Cutting Edge Research (ICCER), Shinshu University, Tokida 3-15-1, Ueda, Nagano 386-8567 Japan; 2 Center of Excellence in Nanotechnology and Materials, Mehran University of Engineering and Technology, Jamshoro, 76060 Sindh Pakistan; 3 Department of Biomedical Engineering, Graduate School of Science and Technology, Shinshu University, Ueda, Nagano 386-8567 Japan

**Keywords:** Nanoscale materials, Structural materials, Structural properties, Synthesis and processing

## Abstract

The use of organic solvents for the preparation of nanofibers are challenged due to their volatile and hazardous behavior. Recently deep eutectic solvents (DES) are widely recognized as non-volatile and non-hazardous solvents which never been utilized directly for nanofabrication via electrospinning. Here, we present the preparation of Zein nanofibers using deep eutectic solvents (DES-Zein). The DES-Zein nanofibers were produced at an optimized polymer concentration of 45% (w/w) with pH 7.3 and electroconductivity 233 mS cm^−1^. DES-Zein nanofibers showed aligned to tweed like cedar leaf morphology tuned by varying the spreading angle from 0° to 90°. In contrast to hydrophobic conventional Zein nanofibers, DES-Zein nanofibers showed super hydrophilic character and about 200 nm finer average diameter. The proposed method of preparing Zein nanofibers using DES opens a new door to continuous electrospinning with tunable morphology, having potential to be used for environmental and biomedical applications.

## Introduction

With a full background of electrospinning development to project the question is  use of  several organic solvents such as Dimethylformamide (DMF)^1^, Chloroform^2^, Trifluoracetic-acid^3^ and Tetra hydro-furan^4^. Electrospinning is developing from fluid blending process to fluid coaxial^1^ and side-by-side^5^, and to tr-fluid coaxial^6^ and other multi-fluid processes^7,8^. However, all these developments are based on the application of organic solvents for preparing working fluids, which is harmful to environment and health^9^.

In contrast, Deep Eutectic Solvents (DES) offers a valuable alternative to overcome the limitations of conventional volatile solvents. DES are widely acknowledged as an ultimate class of Ionic Liquids (ILs) having distinctive chemical properties^10,11^. The prominent applications of DES have initially emerged in the 21st^12^, such as bioengineering^13^, electrochemical synthesis^14^, targeted green extraction^15^, hydrogen transfer^16^, bio-catalysis^17^ and Lithium ion batteries^18^.

The DES based on Quaternary salt (Choline chloride) as a hydrogen bond acceptor (HBA) and (Furfuryl alcohol) as a hydrogen bond donor (HBD) has intensively been in the field of green chemistry allowing a cost-effective synthesis of different green materials and utilization for the extraction of bioactive natural materials^19–23^.

Zein has attracted a great deal of attention due to its biocompatibility, biodegradability, non-toxicity and its wide abundance on earth^18^. Electrospinning of Zein has yet been challenged by the limitation of reusability of the polymer solution and frequent clogging of spinneret when its dissolution occurs in aqueous-ethanol (Aq-EtOH)^24,25^, or its preparation using hazardous solvents, such as DMF^26–28^.

Direct electrospinning of water stable Zein nanofibers have remained a challenging task, due to post-treatments such as longer exposure to Ultraviolet or higher temperatures have been considered as an unavoidable step^29–31^.

Solvents have direct impact on the morphology of resultant fibers and various morphologies of polymeric nanomaterials have been investigated recently, by using different solvent types^32–35^.

The DES have been previously incorporated as a guest molecule used for conveying specific characteristics into chitin nanofibers only, nonetheless direct electrospinning of Zein nanofibers using DES (DES-Zein) has not been reported^36^.

In this article, we attempted to fabricate Zein nanofiber using DES directly by electrospinning technique and achieved super hydrophilic Zein nanofibers without any post-treatment. DES used for electrospinning was based on Choline chloride (HBA) and Furfuryl alcohol (HBD) with a ratio of 1:2 was effectively.

The prepared solution was utilized for the preparation of bead-free Zein nanofibers using 45% (w/w) polymer concentration having the extended shelf life for up to three days. Super hydrophilic DES-Zein nanofibers were compared against the hydrophobic conventional Zein nanofibers (C-Zein) prepared in Aq-EtOH.

DES-Zein nanofibers showed finer average diameter and a unique cedar leaf morphology tuned by varying the spreading angle between the tip and the collector. Precisely, an increase in angle creates alternate coils that leads to a cedar leaf morphology^37–39^.

Super hydrophilicity of C-Zein and DES-Zein nanofibers were confirmed by the wicking test and the water contact angle test^40–42^ and effectively utilized for faster adsorption of impurities present in water^43–45^.

The resultant DES-Zein nanofibers were checked for adsorption capability by removal of the Reactive Black 5 dye. Super hydrophilicity, finer diameter and cedar leaf morphology, all attributed to increased adsorption rate and adsorption capability of DES-Zein nanofibers giving them an edge over C-Zein nanofibers.

The DES-Zein nanofibers can effectively be utilized where faster adsorption and super hydrophilicity is required such as biosensor, biomedical strip, controlled drug release, heavy metal and dye adsorption^46^.

## Results

### Effect of solution properties of C-Zein and DES-Zein on morphology

Solution properties have direct impact on the morphology of nanofibers^47–49^, more specifically polymer ratio, solvent concentration, pH and electro-conductivity (EC) have been studied before electropinning.

EC and pH of DES-Zein and C-Zein polymer solutions were studied by varying the concentrations of Zein in respective polymer solutions, viz. Aq-EtOH, C-Zein, neat DES, DES-Zein 45%, DES-Zein 22.5% and DES-Zein 15% as given in Table 1.

**Table 1:** Solution properties of neat DES, DES-Zein 25%, DES-Zein 35% and DES-Zein 45%, Aq-EtOH and C-Zein.

Solution (10-mL)	Neat DES	DES-Zein 25%	DES-Zein 35%	DES-Zein 45%	Aq-EtOH	C-Zein 25%
pH	4.8	5.6	6.5	7.3	8.0	5.8
mS cm^−1^	1,770	1,380	450	233	5.7	140

Neat DES obtained an EC of 1,770 micro-symon/centimeter (mS cm^−1^) and pH 4.8. An EC of DES-Zein 15% was observed and 1,380 mS cm^−1^ and pH 5.0. DES-Zein 22.5% having an EC of 450 mS cm^−1^ with pH 6.8 and DES-Zein 45% having an EC of 233 mS cm^−1^ with pH 7.3 which is near to neutral pH.

**Figure 1:**
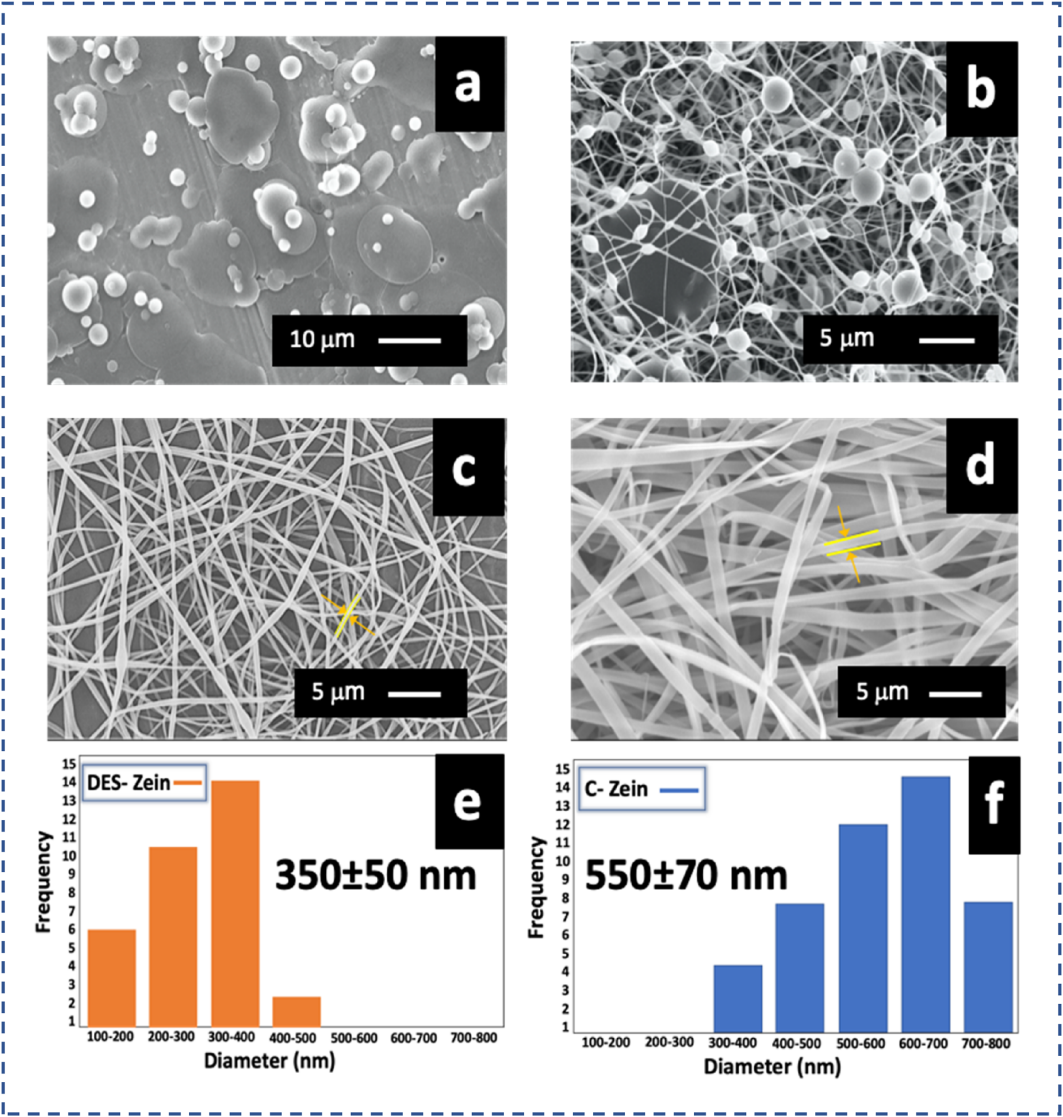
SEM images of (**a**) DES-Z 25%, (**b**) DES-Z 35%, (**c**) DES-Z 45%, (**d**) C-Zein 25%, diameter distribution graphs of (**e**) DES-Zein 45% and (**f**) C-Zein 25%.

The influence of each polymer solution on electrospinnability and their morphology is revealed in Fig. 1. DES-Zein electropun at concentrations (25%, 35% and 45%) with beads, beaded nanofibers and bead-free nanofibers respectively as shown in Fig. 1a–c. On the other hand, Fig. 1d shows bead-free ribbon like morphology of C-Zein nanofibers electrospun at 25% polymer concentration^28^. Figure 1e,f showed average diameter of DES-Zein nanofibers (350 ± 50 nm) and C-Zein nanofibers (550 ± 70 nm).

### Influence of electrospinning parameters on morphology of DES-Zein nanofibers

The quantitative analyses for the fast preparation of nanofibers via electrospinning is totally depended on the basic parameters of the processing which includes, Taylor cone, straight fluid jet and unstable region which are useful for predicting and manipulating a direct link to the quality of resultant nanofibers^48^.

**Figure 2:**
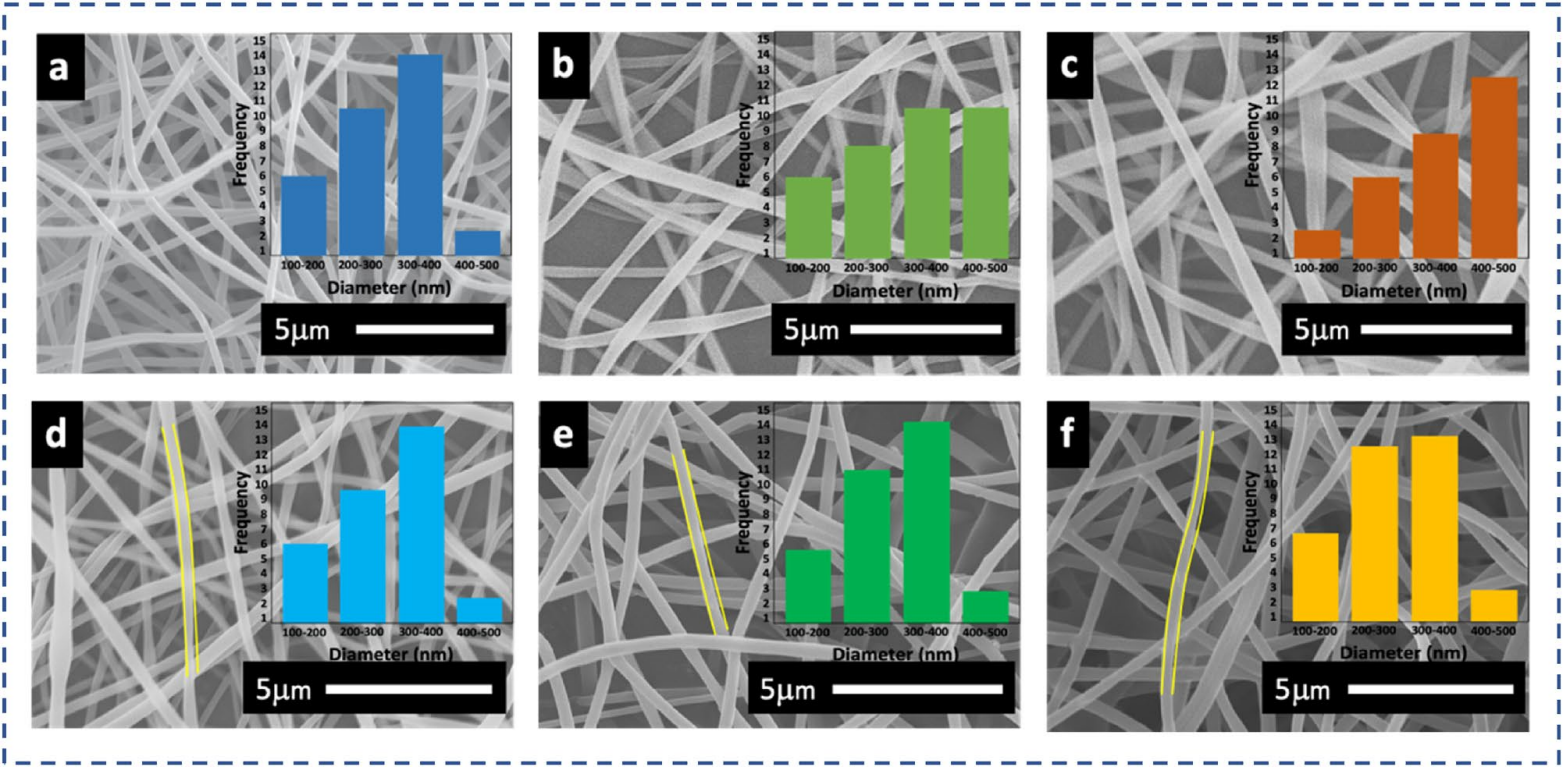
SEM images of DES-Z 45% with voltage 17 kV at tip-collector distance (**a**) 20 cm, (**b**) 14 cm and (**c**) 8 cm, The DES-Z 45% at tip-collector distance 20 cm and voltage (**d**) 10 kV, (**e**) 17 kV and (**f**) 19 kV.

Figure 2 shows the SEM images of dimeter distribution graphs of DES-Zein nanofibers prepared at different tip-collector distances and supplied voltages. Figure 2a–c show the DES-Zein nanofibers prepared at 20 cm, 14 cm and 8 cm tip-collector distance with average diameters 350 ± 50 nm, 400 ± 70 nm and 450 ± 60 respectively, a slight increase in average diameter observed when decreasing the tip-collector distance and minimum diameter distributions observed at maximum distance 20 cm as revealed in the respective inset images, which was optimized to carry further experiments. Whereas Fig. 2d–f show that the supplied voltage has no significant influence on the morphology of DES-Zein nanofibers.

**Figure 3:**
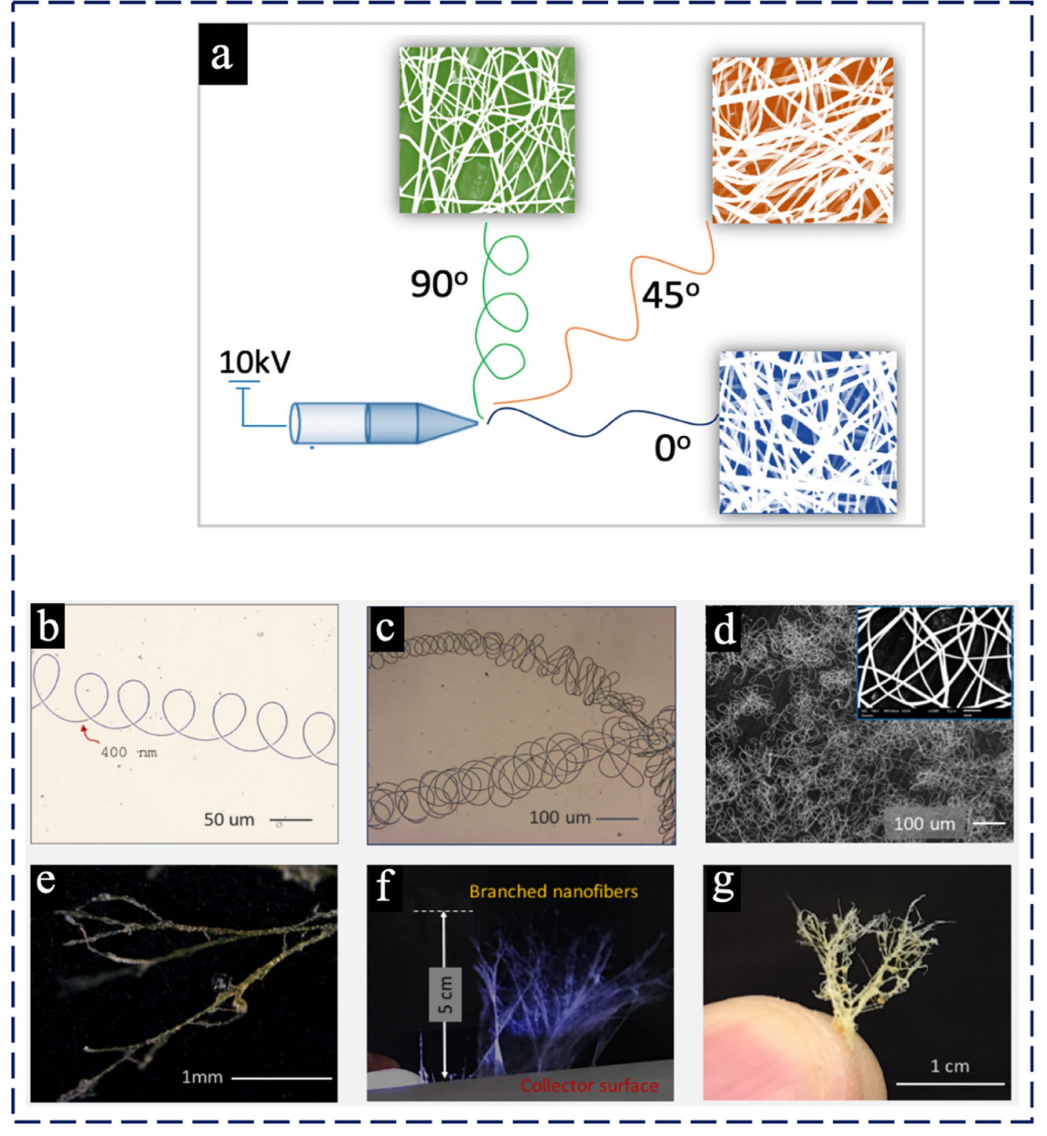
(**a**) Tunability of morphology by varying the spreading angle, microscopic images of (**b**) helical loops, (**c**) multiple alternative helical loops, (**d**) multilayered nanofibers, (**e**) multilayered nanofibers with branches, (**f**) photograph of branched nanofibers growth, (**g**) cedar leaf morphology of DES-Zein.

The spreading angle during electrospinning of DES has a significant influence on nanofiber`s morphology with a very unique and interestingly different than what usually obtained in conventional electrospinning. Therefore, we set different angles at 90°, 45° and 0° to investigate the extent of tunability of nanofibers morphology during electrospinning. Figure 3a illustrates that how nanofiber morphology from straight to coiled can directly be formed by tuning spreading angle from 0° to 90°, coil-less morphology obtained at 0°, semi-coil morphology observed at 45° and at 90° a coiling morphology was achieved. In contrast, the DES-Zein nanofibers, C-Zein did not demonstrate any change in morphology by variation of spreading angle.

Furthermore, Fig. 3b shows the coiling configuration of coil shaped morphology of DES-Zein nanofibers having a gap of 85 ± 5 μm between two neighboring coils. Figure 3c shows microscopic image of the consistent coil formation in different directions from a divergent point ultimately resulting a cedar leaf effect. Figure 3d shows coil shaped nanofibers collected on a stationary collector. Figure 3e shows the image of electrospun DES-Zein nanofibers over a stationery metallic collector that resulted in a “tweed like” structure and further prolonged corrugation of four hours resulted into a 3D cedar leaf morphology as shown in Fig. 3f,g. The probable reason for coils formation during electrospinning was due to α-helix of amino acid series present in the native Zein, the DES with the hydrogen bonding assistance actually preserves the α-helix configuration of Zein during electrospinning of DES-Zein nanofibers. Another reason for coil formation may be the higher conductivity of DES due to the presence of Choline chloride as a salt that may create gaps at the perpendicular collector angle for electrospinning^28,29^.

### Chemical structure of C-Zein and DES-Zein

**Figure 4:**
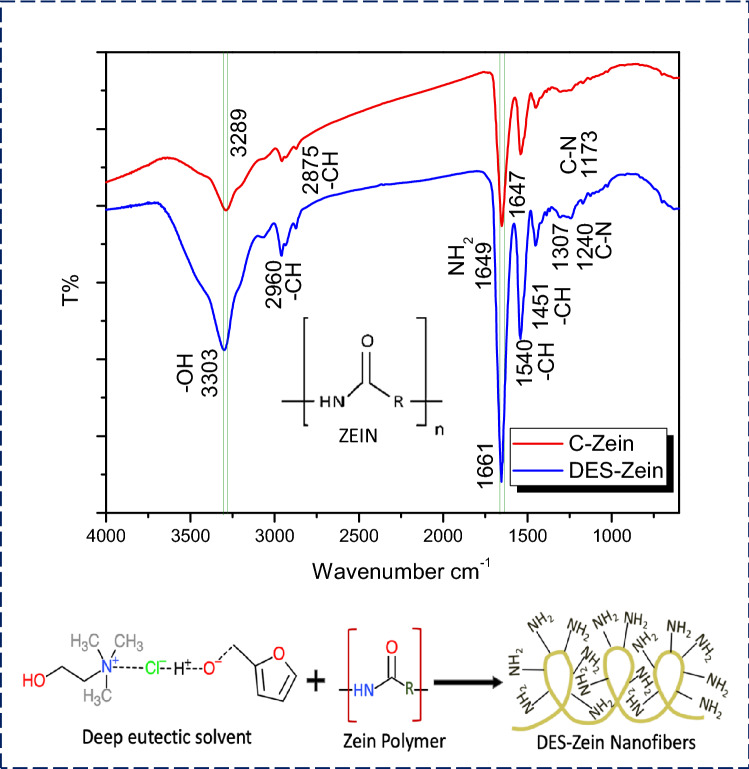
FTIR spectrum of C-Zein and DES-Zein with proposed chemical structure.

Figure 4 shows the FTIR spectrum of electrospun C-Zein and DES-Zein nanofibers. C-Zein showed a significant peak at 3,289 cm^−1^ due to –NH stretching, while DES-Zein showed intensive and broad stretching peak at 3,303 cm^−1^ due to the strong hydrogen bonding between –OH from Choline chloride/furfuryl-alcohol moiety and –NH from Zein polymer^15,16,22^.

The intense bands at 2,875 cm^−1^ and 2,960 cm^−1^ due to the asymmetric and symmetric C–H stretching in the spectrum of DES-Zein nanofibers, which may be due to the induced dipole moment caused by the change in solvent polarity comparing to C-Zein nanofibers. Peaks at 1,647 cm^−1^ and 1,661 cm^−1^ clearly show the amide I (C=O) stretching, the bands at 1,540 cm^−1^ in both C-Zein and DES-Zein may be due to presence of amide II (angular deformation vibration of –NH bond).

The band at 1,240 cm^−1^ in DES Zein and 1,170 cm^−1^ in C-Zein reveal the axial deformation of C–N vibration and no peaks found in C-Zein at 1,662 cm^−1^, 1,631 cm^−1^ and 1,614 cm^−1^.

Zein nanofibers obtained either from a conventional solvent or a DES, do not have any profound difference in chemical composition as shown in Supplementary Fig. S1a, wide scan XPS spectrum of electropun nanofibers was obtained for both samples (C-Zein and DES-Zein), which contain C1s (284 eV), N1s (399 eV) and O1s (530 eV). Peak fitting of every component showed a similar composition as C-Zein nanofibers obtained from conventional solvents^28^.

Supplementary Fig. S1b shows the deconvolution of C1s peak from DES-Zein revealing three main components C–C, C–N and O=C–N at 284 eV, 285 eV and 287 eV respectively. Supplementary Fig. S1c shows the deconvolution of O1s indicating two different peaks at 529.3 eV and 529.9 eV indicating the presence of O–C and O=C–N respectively. Furthermore, Supplementary Fig. S1d shows the deconvolution of N1s revealing the presence of N in three chemical states N–C, N–C=O and –NH at 398 eV, 399 eV and 400 eV, respectively. This finding suggests that the DES solvent do not bring any substantial changes to the chemical composition of Zein polymer.

### Comparison of crystallinity between DES-Zein and C-Zein nanofiber

**Figure 5:**
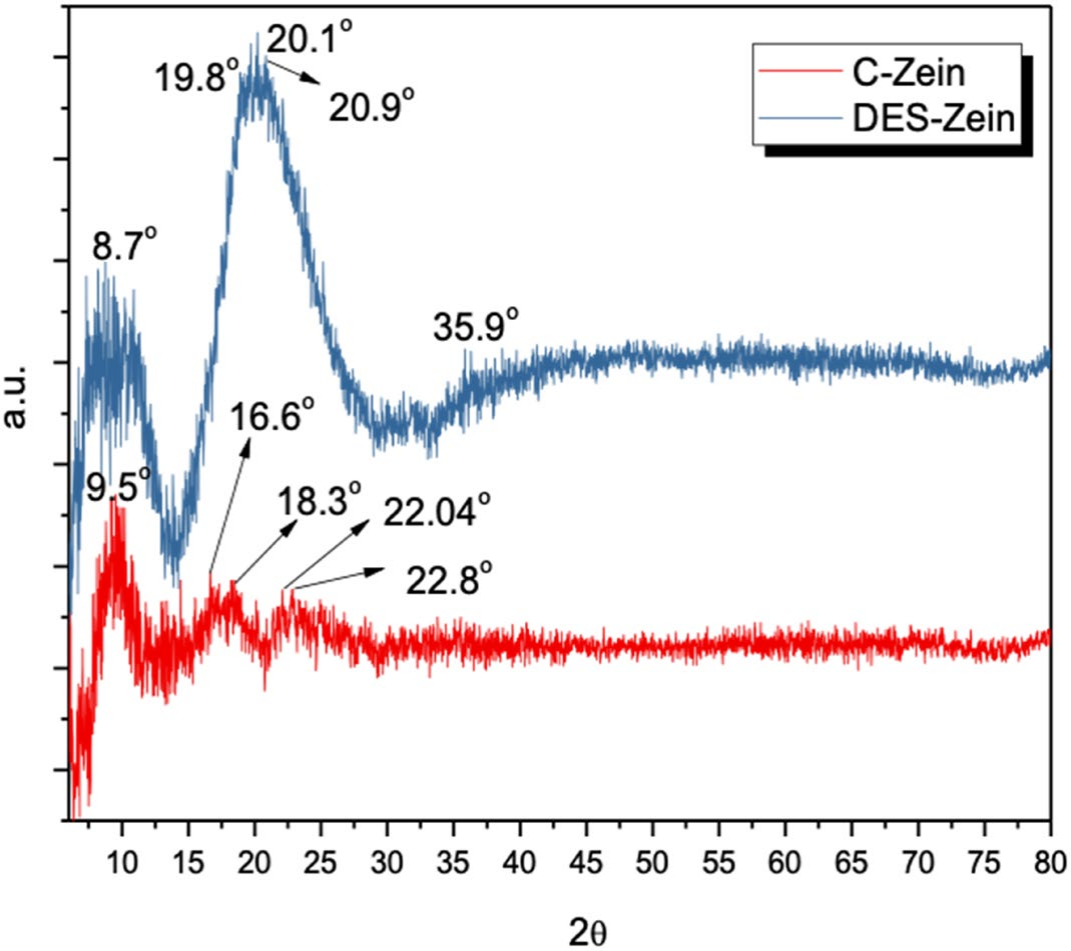
XRD Comparison of C-Zein and DES-Zein.

Figure 5 shows the XRD patterns of C-Zein and DES-Zein nanofibers. C-Zein shows intensive peak at 9.5^o^ with d-spacing (9.3 Å) and some small peaks at angles 16.6°,18.3°, 22.04°, 22.8° with d-spacing (5.3 Å), (4.8 Å), (4.0 Å), (3.8 Å) respectively, whereas DES-Zein shows intensive peaks at 8.7° with d-spacing (10.2 Å ) and some highly intensive peaks at 19.8°, 20.1°, 20.9° with d-spacing (4.48 Å), (4.42 Å), (4.2 Å) respectively and an additional small peak also observed at 35.9° with d-spacing (2.4 Å).

### Hydrophilicity of DES-Zein nanofibers

**Figure 6:**
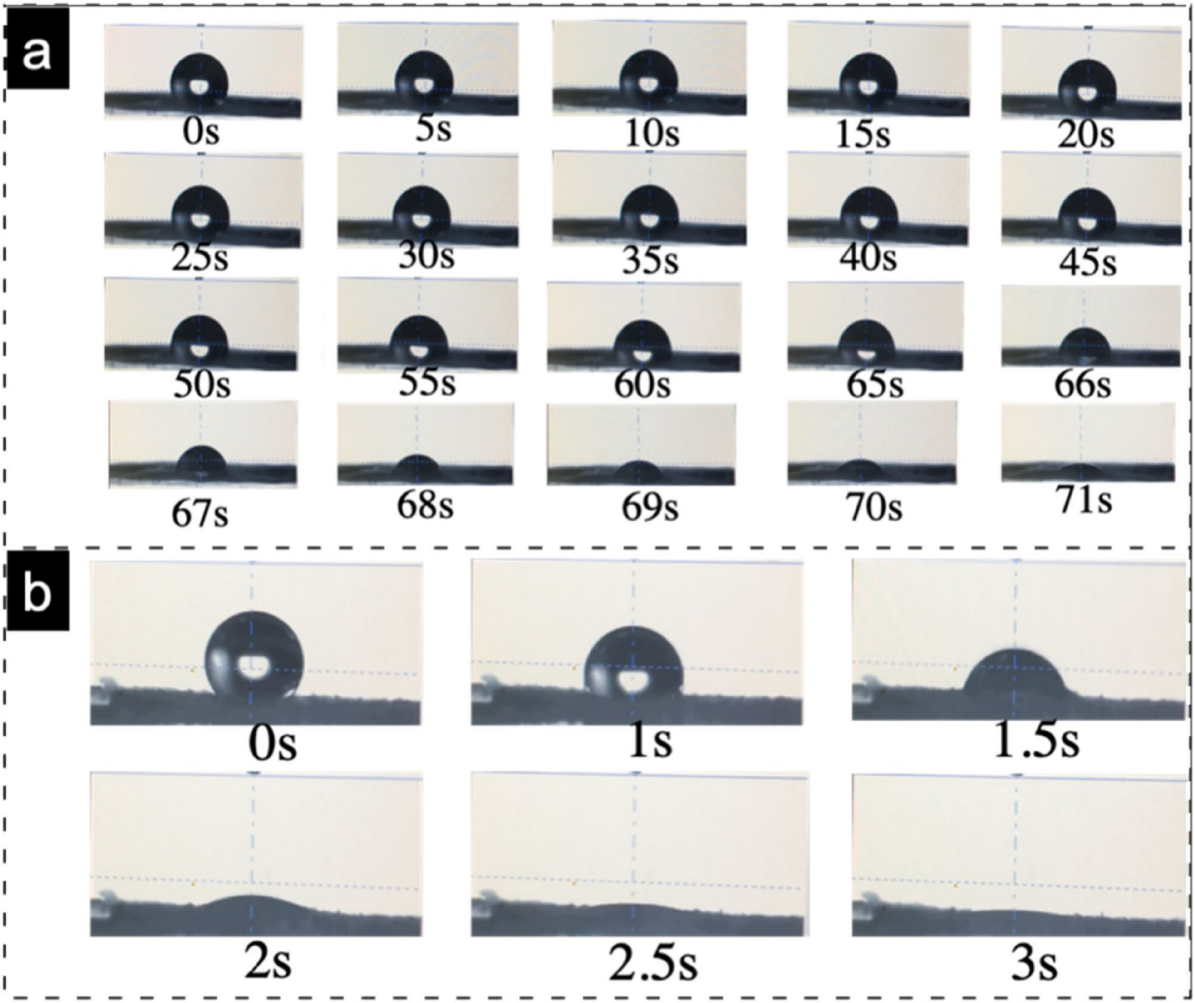
Water contact angle of (**a**) C-Zein and (**b**) DES-Zein.

Water contact angle (WCA) of C-Zein and DES-Zein is demonstrated in Fig. 6. The WCA of in Fig. 6a shows, C-Zein takes 70 ± 3 s to reach the angle from 115° ± 10° to 2° ± 2°, whereas, Fig. 6b shows that DES-Zein takes only 3 ± 1 s to decrease in angle from 122° ± 10° to 0°.

**Figure 7:**
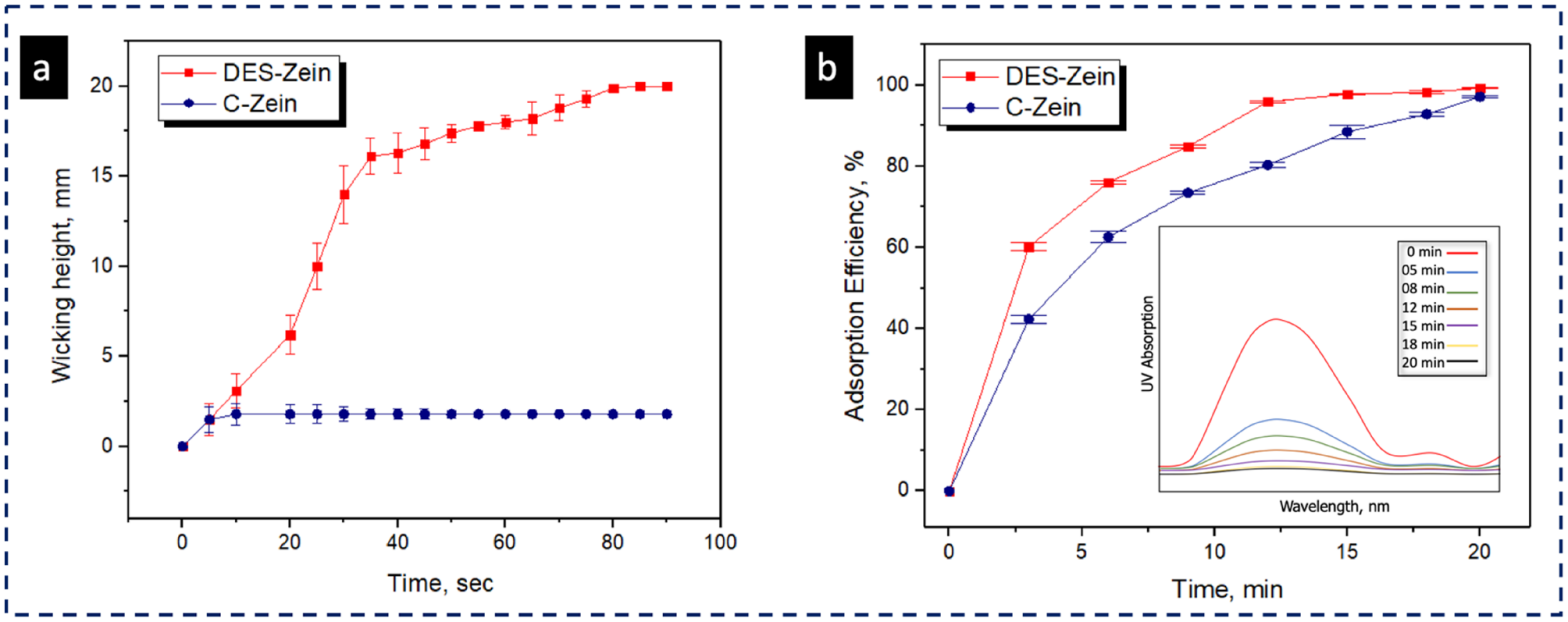
(**a**) Wicking profile of DES-Zein and C-Zein (**b**) adsorption efficiency graph with inset UV–Vis profile.

Hydrophobic behavior of C-Zein and super hydrophilic behavior of DES-Zein nanofibers further confirmed by rate of wicking as shown in Fig. 7a. C-Zein shows the wicking height of 2 mm at initial dipping into dye solution and no more wicking observed until 90 s. However, DES-Zein reached the wicking height of > 16 mm in first 30 s and slowly reached to the wicking height of 20 mm within 75 s, confirming the super hydrophilic behavior of DES-Zein nanofibers.

### Dye adsorption capability of DES-Zein nanofibers

Figure 7b shows the dye removal efficiency of DES-Zein nanofibers. Reactive black 5 dye was used to check the dye removal capability of DES-Zein nanofibers because C-Zein has been previously well studied for selective removal of reactive black 5 dye^28^. This study reveals UV–Vis spectrophotometry in which absorbance values of dye solutions were individually checked at different time intervals after dye adsorption on DES-Zein. Increase in dye adsorption on DES-Zein was the result of decreased absorption peaks of UV spectrophotometer with respect to time. DES-Zein nanofibers have capability to remove 60% of the dye within first 3 min, whereas the C-Zein nanofiber have a slower removal capability with 45% in the same time, which confirms the rapid adsorption of DES-Zein.

The removal efficiency of DES-Zein observed 98.29 ± 1% within 18 min and (99 ± 1) % in 20 min, whereas the removal efficiency of C-Zein observed 97.19 ± 1% in 20 min which is lesser than the removal efficiency of DES-Zein achieved within 18 min. Increased dye removal efficiency was achieved by DES-Zein at pH 3.9–7.3 as compared to the dye removal efficiency C-Zein at pH 2–6.

## Discussion

DES can directly be utilized for electrospinning of Zein at optimized concentration 45% (w/w) at pH 7.3 having an EC of 233 mS cm^−1^, offering the solution stability for further electrospinning up to three days. The EC values decreased with increasing Zein concentration into DES and the pH was increased due to the protonation of Zein in acidic medium (DES) which can be stabilized through salt bridges of Choline chloride.

On contrary, the addition of Zein polymer into Aq-EtOH resulted increased EC 140 mS cm^−1^ and decreases pH in comparison to the Aq-EtOH solution, the obvious reasons for this is because of the less free hydrogens of C-Zein and polyelectrolytic behavior of Aq-EtOH after solubilizing Zein polymer in it^33,50^.

No difference in DES-Zein nanofiber morphology was observed by varying voltage supply of 10–19 kV during electrospinning, above 19 kV the charges induced were so high that did not allow the polymer solution to pass through the tip, therefore no nanofiber formation observed. Therefore, the lowest 10 kV was selected as optimized voltage for electrospinning of DES-Zein, on the other hand preparation of C-Zein in Aq-EtOH requires voltage supply of 22–25 kV for nanofabrication which is quite higher than the voltage required for preparation of DES-Zein nanofibers.

The average diameter of DES-Zein nanofibers (350 ± 50 nm) was observed as around 200 nm finer than the average diameter of C-Zein nanofibers (550 ± 70 nm). Also, in contrast to cedar leaf tunable morphology of DES-Zein nanofibers, C-Zein did not demonstrate any change in morphology by variation of spreading angle.

The basic reason for unique morphology was helices and branched coil formation of DES-Zein during electrospinning at angle 90°, the probable reason for coils formation during electrospinning was due to α-helix of amino acid series present in the native Zein, the deep eutectic solvent with the hydrogen bonding assistance actually preserves the α-helix configuration of Zein during electrospinning of DES-Zein nanofibers.

Another reason for coil formation may be the higher conductivity of DES due to the presence of Choline chloride as a salt that may create gaps at the perpendicular collector angle for electrospinning^34,35^.

The chemical spectra of FTIR also proposed some chemical changes of protonation of Zein due to inter/intra hydrogen bonding between –NH groups in the Zein polymer when dissolved in DES^51^. No peak was found in C-Zein at 1662 cm^−1^, 1631 cm^−1^ and 1614 cm^−1^ which clearly reveals a major presence of α helices in DES-Zein rather than β sheets or β turns^52^. α helices in the chemical structure of DES-Zein can be one of the reasons for the tweed-like morphology formed during electrospinning.

Evidently, DES-Zein showed increased crystallinity compared to C-Zein due the bulk presence of –OH bonds as supported by FTIR results, which was the reason of decreased distance between the molecules in the molecular chain and increased diffraction angles^52^. The d-spacing of ~ 4.5 Å is considered to be the average backbone distance within the α-helix of Zein structure. However, in parallel the larger d-spacing of ~ 10 Å is believed to be the spacing of the inter-helix packing or the mean distance approach to the neighboring helices^50,52^.

The increase in its intensity implies the irruption of DES-Zein molecular aggregation because of increased d-spacing and more inter-helix packing of DES-Zein^47^. The faster adsorption achieved by DES-Zein was due to the electrostatic interactions between the –NH and –OH and their abundancy on surface of nanofibers resulted from the protonation by DES^53^.

WCA and wicking confirmed the super hydrophilic nature of DES-Zein nanofibers in contrast to hydrophobic conventional Zein, due to presence of more amines and hydrogen bonds on the surface of ribbons, that was the reason that DES-Zein nanofibers showed faster and increased dye removal efficiency, Hence, DES-Zein can potentially be considered for the applications where faster adsorption is required such as biosensors and selective adsorption of impurities present in environment.

## Conclusion

Zein nanofibers were successfully electrospun using DES. The optimized parameters to produce bead free nanofibers were 45% (w/w) polymer concentration with pH 7.3 and EC 233 mS cm^−1^. The resultant DES-Zein nanofibers showed aligned to tweed like cedar leaf morphology tuned by varying the spreading angle from 0° to 90°. The average diameter of DES-Zein nanofibers was 350 ± 50 nm, about 200 nm finer than the average diameter of C-Zein nanofibers 550 ± 70 nm. In contrast to hydrophobic C-Zein nanofibers, DES-Zein nanofibers showed super hydrophilic character confirmed by WCA, the drop from 122° ± 10° promptly sunk to 0° within 3 s. Additionally, DES-Zein nanofiber showed higher wicking rate, thanks to the cedar leaf morphology and the abundant presence of –NH and –OH groups on the surface of DES-Zein nanofibers, which allow it to be used where super hydrophilicity is required.

## Materials and methods

Zein produced from maize having a melting point of (266–283 °C), Choline chloride (> 98%) with molecular weight 139.62 g mol^−1^ and Furfuryl alcohol (98%) with molecular weight 98.10 g mol^−1^ were supplied by Sigma-Aldrich, USA. Ethanol (99.8%) with molecular weight 46.07 g mol^−1^ was supplied by Merck, Japan. CI reactive black 5 bis(sulphatoethylsulphone) (Mw = 991.82 g mol^−1^) purchased from Sumitomo Chemical Co., Ltd., Japan.

### Preparation of DES-Zein nanofibers

Zein Nanofibers were prepared separately in conventional and deep eutectic solvent using (Har-100*12, Matsusada Co., Tokyo, Japan) Electrospinning machine. Zein 25% (w/v) in 80% of Aq-EtOH was stirred for 2 h at 80 °C^21^. The DES was prepared by mixing Choline chloride and Furfuryl alcohol with molar ratio 2:1 and stirred well until a clear transparent solution was obtained. Zein polymer was then added into DES solution with different concentrations (20, 25, 30, 35, 40, 45, 50 and 55% w/w) and well stirred up to 2 h at 80 °C. Each electrospinning solution was poured into a syringe of 5 mL attached to a tip of 0.6 mm internal diameter and the flow rate was set to 0.2 mL h^−1^. Copper wire was used as an anode dipped into the Zein solution, and the ground collector was connected as a cathode. The electrospinning parameters were optimized to produce uniform and consistent DES-Zein nanofibers. The voltages supplied for electrospinning of DES-Zein and C-Zein were optimized as 10 kV and 25 kV respectively and the distances between the tip and the collector for DES-Zein and C-Zein were optimized as 14 cm and 17 cm respectively. The DES-Zein nanofibers were consistently deposited on a stationary collector at different angles between the tip and the collector from 0° to 90°. After electrospinning, the obtained samples were dried overnight at room temperature to remove residual solvents before subjecting to other characterizations.

### Characterizations

Polymer solution properties of DES-Zein such as pH and conductivity were assessed using (WTW MultiLine IDS multi-parameter portable meter). The surface morphology examined under SEM (S-3000N by Hitachi, Japan), C-Zein and DES-Zein were sputtered with Pb–Pt under vacuumed environment prior to take SEM images and the average diameter of was calculated using ImageJ software.

The chemical structure of DES-Zein was assessed through Fourier Transform Infrared (FTIR) spectroscopy (IR Prestige-21 by Schimadzu, Japan) using ATR mode and Xray Photoelectron spectroscopy (XPS) by AXIS Ultra (Schimadzu) with dual-anode X-ray source Al/Mg, HSA hemispherical sector analyzer and the detector, vacuum pressure was maintained at 1.4 × 10^–9^ torr, and Mg Kα X-ray source (1,253.6 eV) was used.

Wide-angle Xray diffractometer (WAXD) Rigaku Miniflex 300 from Japan was used to compare the crystallinity of C-Zein and DES-Zein where the range was 10–80° at the scanning speed 2θ = 2° min^−1^. The WCA test was carried on FACE model CA-VP, Kyowa interface science, Japan, five readings were assessed from each sample. The wicking behavior of DES-Zein nanofibers was checked by previously reported methods^35–37^.

### Adsorption studies

The residual values of reactive black 5 after adsorption on DES-Zein nanofibers were analyzed by using Lambda 35 UV–Vis spectrophotometer from Perkin Elmer USA. The absorbance values were taken at λ_max_ 590 nm. The average-thickness of DES-Zein nanofibers was 35 μm. The DES-Zein nanofiber samples were checked for dye adsorption capability, 50 g L^−1^ Reactive black 5 was used to check the dye adsorption on novel DES-Zein. The mass 40 ± 0.2 mg of DES-Zein nanofibers was shacked in glass tube filled with 5 mL of Reactive black 5 dye solution at room temperature. Dye adsorption values were then checked at different intervals (0, 5, 8, 12, 15, 18, 20, and 25) min^44,45^.

## Supplementary information

Supplementary Figure 1

## References

[1] Zhou X, Jin W, Sun C, Gao SH, Chen C, Wang Q, Wang Q (2018). Microbial degradation of N, N-dimethylformamide by Paracoccus sp. strain DMF-3 from activated sludge. Chem. Eng. J..

[2] Baskar K, Ananthi J, Ignacimuthu S (2018). Toxic effects of *Solanum xanthocarpum* Sch & Wendle against *Helicoverpa armigera* (Hub.), *Culex quinquefasciatus* (Say.) and *Eisenia fetida* (Savigny, 1826). Environ. Sci. Pollut. Res..

[3] Viglianisi C, Scarlini A, Tofani L, Menichetti S, Baschieri A, Amorati R (2019). Magnetic nanoantioxidants with improved radical-trapping stoichiometry as stabilizers for inhibition of peroxide formation in ethereal solvents. Sci. Rep..

[4] Zhou H, Shi Z, Wan X, Fang H, Yu DG, Chen X, Liu P (2019). The relationships between process parameters and polymeric nanofibers fabricated using a modified coaxial electrospinning. Nanomaterials.

[5] Yu DG, Li JJ, Zhang M, Williams GR (2017). High-quality Janus nanofibers prepared using three-fluid electrospinning. Chem. Commun..

[6] Zhao K, Wang W, Yang Y, Wang K, Yu DG (2019). From Taylor cone to solid nanofiber in tri-axial electrospinning: Size relationships. Results Phys..

[7] Riley, R. S., & Day, E. S. *Wiley Interdiscip*. *Rev. Nanomed. Nanobiotechnol* 9(4): 1449 (2017). 10.1002/wnan.1449 PMC5474189 28160445

[8] Yu DG, Wang M, Li X, Liu X, Zhu LM, Annie Bligh SW (2019). Multifluid electrospinning for the generation of complex nanostructures. Nanomed. Nanobiotechnol. Wiley Interdiscip. Rev..

[9] Smith EL, Abbott AP, Ryder KS (2014). Deep eutectic solvents (DESs) and their applications. Chem. Rev..

[10] Nkuku CA, LeSuer RJJ (2007). Electrochemistry in deep eutectic solvents. J. Phys. Chem. B.

[11] Paiva A, Craveiro R, Aroso I, Martins M, Reis RL, Duarte ARC (2014). Natural deep eutectic solvents–solvents for the 21st century. ACS Sustain. Chem. Eng..

[12] Mbous YP, Hayyan M, Hayyan A, Wong WF, Hashim MA, Looi CY (2017). Applications of deep eutectic solvents in biotechnology and bioengineering—promises and challenges. Biotechnol. Adv..

[13] Abo-Hamad A, Hayyan M, AlSaadi MA, Hashim MA (2015). Potential applications of deep eutectic solvents in nanotechnology. Chem. Eng. J..

[14] Chatterjee M, Ishizaka T, Kawanami H (2018). Accelerated decarbonylation of 5-hydroxymethylfurfural in compressed carbon dioxide: A facile approach. Green Chem..

[15] Paulino PN, Perez RF, Figueiredo NG, Fraga MA (2017). Tandem dehydration–transfer hydrogenation reactions of xylose to furfuryl alcohol over zeolite catalysts. Green Chem..

[16] Millia L, Dall'Asta V, Ferrara C, Berbenni V, Quartarone E, Perna FM, Mustarelli P (2018). Bio-inspired Choline chloride-based deep eutectic solvents as electrolytes for lithium-ion batteries. Solid State Ion..

[17] Wang Y, Niu Z, Zheng Q, Zhang C, Ye J, Dai G, Zhang X (2018). Zn-based eutectic mixture as anolyte for hybrid redox flow batteries. Sci. Rep..

[18] Li G, Li N, Zheng M, Li S, Wang A, Cong Y, Zhang T (2016). Industrially scalable and cost-effective synthesis of 1, 3-cyclopentanediol with furfuryl alcohol from lignocellulose. Green Chem..

[19] Perez RF, Fraga MA (2014). Hemicellulose-derived chemicals: One-step production of furfuryl alcohol from xylose. Green Chem..

[20] Nam MW, Zhao J, Lee MS, Jeong JH, Lee J (2015). Enhanced extraction of bioactive natural products using tailor-made deep eutectic solvents: Application to flavonoid extraction from Flos sophorae. Green Chem..

[21] Abbott AP, Cihangir S, Ryder KS (2018). Redox fusion of metal particles using deep eutectic solvents. Chem. Commun..

[22] Wang HJ, Cao Y, Wang CF, Cui SZ, Mi LW, Miyazawa T (2016). Green self-assembly of zein-conjugated ZnO/Cd (OH) Cl hierarchical nanocomposites with high cytotoxicity and immune organs targeting. Sci. Rep..

[23] Lu M, Han G, Jiang Y, Zhang X, Deng D, Ai N (2015). Solubilities of carbon dioxide in the eutectic mixture of levulinic acid (or furfuryl alcohol) and Choline chloride. J. Chem. Thermodyn..

[24] Wang M, Hai T, Feng Z, Yu DG, Yang Y, Annie Bligh SW (2019). The relationships between the working fluids, process characteristics and products from the modified coaxial electrospinning of zein. Polymers.

[25] Wang Q, Yu DG, Zhang LL, Liu XK, Deng YC, Zhao M (2017). Electrospun hypromellose-based hydrophilic composites for rapid dissolution of poorly water-soluble drug. Carbohyd. Polym..

[26] Tomé LI, Baião V, da Silva W, Brett CM (2018). Deep eutectic solvents for the production and application of new materials. Appl. Mater. Today.

[27] Amiri N, Moradi A, Tabasi SAS, Movaffagh J (2018). Modeling and process optimization of electrospinning of chitosan-collagen nanofiber by response surface methodology. Mater. Res. Express.

[28] Qureshi UA, Khatri Z, Ahmed F, Khatri M, Kim IS (2017). Electrospun zein nanofiber as a green and recyclable adsorbent for the removal of reactive black 5 from the aqueous phase. ACS Sustain. Chem. Eng..

[29] Yao C, Li X, Song T (2007). Electrospinning and crosslinking of zein nanofiber mats. J. Appl. Polym. Sci..

[30] Lu H, Wang Q, Li G, Qiu Y, Wei Q (2017). Electrospun water-stable zein/ethyl cellulose composite nanofiber and its drug release properties. Mater. Sci. Eng. C.

[31] Woods GW, Sessa KK, Biswas A (2008). Electrospun zein fibers using glutaraldehyde as the crosslinking reagent: Effect of time and temperature. Macromol. Chem. Phys..

[32] Anbukarasu P, Sauvageau D, Elias A (2015). Tuning the properties of polyhydroxybutyrate films using acetic acid via solvent casting. Sci. Rep..

[33] Samuel C, Raquez JM, Dubois P (2013). PLLA/PMMA blends: A shear-induced miscibility with tunable morphologies and properties. Polymer.

[34] Ravati S, Favis BD (2013). Tunable morphologies for ternary blends with poly (butylene succinate): Partial and complete wetting phenomena. Polymer.

[35] Zhang X, Zhu J, Haldolaarachchige N, Ryu J, Young DP, Wei S, Guo Z (2012). Synthetic process engineered polyaniline nanostructures with tunable morphology and physical properties. Polymer.

[36] Mukesh C, Mondal D, Sharma M, Prasad K (2014). Choline chloride–thiourea, a deep eutectic solvent for the production of chitin nanofibers. Carbohyd. Polym..

[37] Du C, Zhao B, Chen XB, Birbilis N, Yang H (2016). Effect of water presence on Choline chloride-2urea ionic liquid and coating platings from the hydrated ionic liquid. Sci. Rep..

[38] Yao T, Li X, Lin Q, Jie W, Ren Z, Wang C, Yang B (2009). Patterns of conducting polypyrrole with tunable morphologies. Polymer.

[39] Huan W, Zhang J, Qin H, Huan F, Wang B, Wu M, Li J (2019). A magnetic nanofiber-based zwitterionic hydrophilic material for the selective capture and identification of glycopeptides. Nanoscale.

[40] Ahmed F, Arbab AA, Jatoi AW, Khatri M, Memon N, Khatri Z, Kim IS (2017). Ultrasonic-assisted deacetylation of cellulose acetate nanofibers: A rapid method to produce cellulose nanofibers. Ultrason. Sonochem..

[41] Khatri M, Ahmed F, Jatoi AW, Mahar RB, Khatri Z, Kim IS (2016). Ultrasonic dyeing of cellulose nanofibers. Ultrason. Sonochem..

[42] Khatri M, Ahmed F, Shaikh I, Phan DN, Khan Q, Khatri Z, Lee H, Kim IS (2017). Dyeing and characterization of regenerated cellulose nanofibers with vat dyes. Carbohyd. Polym..

[43] Zang L, Lin R, Dou T, Ma J, Sun L (2019). Electrospun superhydrophilic membranes for effective removal of Pb (II) from water. Nanosc. Adv..

[44] Qureshi UA, Khatri Z, Ahmed F, Ibupoto AS, Khatri M, Mahar FK, Brohi RZ, Kim IS (2017). Highly efficient and robust electrospun nanofibers for selective removal of acid dye. J. Mol. Liq..

[45] Ibupoto AS, Qureshi UA, Ahmed F, Khatri Z, Khatri M, Maqsood M, Brohi RZ, Kim IS (2018). Reusable carbon nanofibers for efficient removal of methylene blue from aqueous solution. Chem. Eng. Res. Des..

[46] Yang Y, Zhu T, Liu Z, Luo M, Yu DG, Bligh SA (2019). The key role of straight fluid jet in predicting the drug dissolution from electrospun nanofibers. Int. J. Pharm..

[47] Morris MC, McMurdie HF, Evans EH, Paretzkin B, Parker HS, Panagiotopoulos NC, Hubbard CR (1981). Standard X-ray diffraction powder patterns, NBS monograph 25, Section 18, data for 58 substances. Gaithersburg Natl. Bureau Std..

[48] Son WK, Youk JH, Lee TS, Park WH (2004). The effects of solution properties and polyelectrolyte on electrospinning of ultrafine poly (ethylene oxide) fibers. Polymer.

[49] Shen L, Huang X (2019). Tuning the morphologies and electrical properties of azobenzene-4, 4′-dicarboxylate-doped polypyrrole via ultraviolet light irradiation and medium pH alteration. Polymer.

[50] Ali S, Khatri Z, Oh KW, Kim IS, Kim SH (2014). Zein/cellulose acetate hybrid nanofibers: Electrospinning and characterization. Macromol. Res..

[51] Shi K, Kokini JL, Huang Q (2009). Engineering zein films with controlled surface morphology and hydrophilicity. J. Agric. Food Chem..

[52] Mejia CD, Gonzalez DC, Mauer LJ, Campanella OH, Hamaker BR (2012). Increasing and stabilizing β-sheet structure of maize zein causes improvement in its rheological properties. J. Agric. Food Chem..

[53] Erdogan I, Demir M, Bayraktar O (2015). Olive leaf extract as a crosslinking agent for the preparation of electrospun zein fibers. J. Appl. Polym. Sci..

