# Supplementary material for: Zein nanofibers via deep eutectic solvent electrospinning: tunable morphology with super hydrophilic properties
Source: Sci Rep. 2020 Sep 17;10:15307. doi: 10.1038/s41598-020-72337-4 (PMC7499184; doi:10.1038/s41598-020-72337-4)
Supplement: Supplementary file 1 — Supplementary Figure 1 [file 41598_2020_72337_MOESM1_ESM.docx]

**Zein nanofibers via deep eutectic solvent electrospinning: Tunable morphology with super hydrophilic properties**

Muzamil Khatri1, Zeeshan Khatri1,2*, Sofia El-Ghazali3, Nadir Hussain1, Umair Ahmed Qureshi2, Shunichi Kobayashi3, Farooq Ahmed2, Ick Soo Kim1*


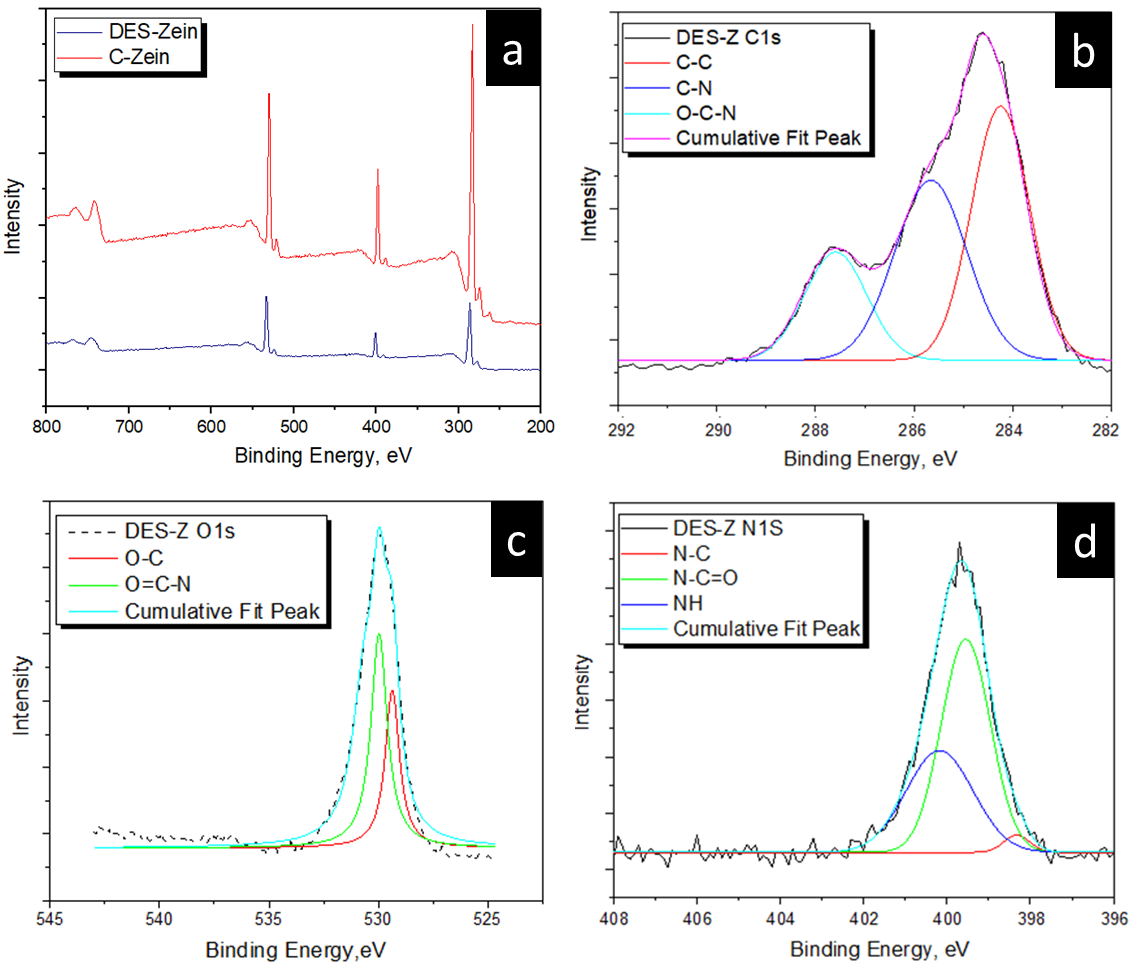


**Fig. S1.** (a) Wide XPS spectrum of DES-Zein and C-Zein, XPS spectra of (b) DES-Zein C1s, (c) DES-Zein O1s and (d) DES-Zein N1s
